# Leveraging Technology and Gamification to Engage Learners in a Microbiology Curriculum in Undergraduate Medical Education

**DOI:** 10.1007/s40670-022-01552-7

**Published:** 2022-05-04

**Authors:** Jeremey Walker, Jose Pablo Heudebert, Mukesh Patel, John D. Cleveland, Andrew O. Westfall, Donald M. Dempsey, Alfredo Guzman, Anne Zinski, Monica Agarwal, Dustin Long, James Willig, Rachael Lee

**Affiliations:** 1 Department of Medicine, School of Medicine (UAB), University of Alabama, THT 229, 1900 University Blvd, AB 35294-0006 Birmingham, USA; 2 University of Alabama School of Medicine, Birmingham, AL USA; 3 Birmingham Veterans Affairs Medical Center, Birmingham, USA; 4 Department of Biostatistics, University of Alabama at Birmingham, Birmingham, USA; 5 Department of Microbiology, University of Alabama at Birmingham, Birmingham, USA; 6 Department of Medical Education, University of Alabama School of Medicine, Birmingham, USA

**Keywords:** Undergraduate medical education (UME), Gamification, Microbiology, Curriculum

## Abstract

**Background:**

Microbiology is a critical and expansive topic that many medical schools’ curriculum must teach in a constrained time frame. We implemented a microbiology question bank smart phone app enhanced with game elements and clinical pearls during a microbiology course for first-year medical students. We hypothesized that these enhancements and clinical pearls would engage the students meaningfully and increase their knowledge base.

**Methods:**

Though use was optional, students’ game play was recorded through the app, which was compared to test grades retrospectively. A player efficiency rating (PER) was calculated as a function of question response, accuracy, and engagement. Students were separated into tertiles of PER and median exam grades were compared using a non-parametric Kruskal–Wallis (KW) test. An anonymous satisfaction and usability feedback survey was also administered.

**Results:**

One hundred eighty-one of the 189 students (96%) answered at least one question, and 165 (87%) completed all 56 questions. The average PER was 84.75. We received feedback surveys from 61 (34%) students in the course, with positive responses regarding the perceived impact on learning microbiology. The KW test found a positive correlation for median exam scores of the player groups when divided into tertiles by PER (*p* = 0.0002).

**Conclusions:**

We leveraged gamification and clinical pearls to design a supplemental microbiology question bank. We found high engagement overall and higher class exam scores associated with greater use of the question bank.

## Introduction

Microbiology is the first exposure for medical students to the field of clinical infectious diseases (ID), and remains the roots from which this branch of medicine often flowers. As undergraduate medical education (UME) has evolved, competing demands on limited medical school curricular time have increased the constraints on teaching time nationally [1, 2]. At our medical school, we saw the reflection of these national trends in student evaluations in which the microbiology course was reported as challenging due to a large quantity of material condensed into a short course, and limited time to form clinical correlations. These educational developments are concerning, as pre-clerkship microbiology courses are a key opportunity to influence students to select a career path in infectious diseases and are foundational for the practice of medicine [3, 4]. A survey of microbiology course directors in 2016 found technology-enhanced learning modalities and increasing clinical relevance of material as innovative themes in microbiology education [1].

The last decade has seen an increase in technology utilization within UME, particularly in pre-clerkship years with programs such as UWorld, Kaplan, and Boards and Beyond that provide a question bank and multimedia platform focused on core content that is felt to be relevant for board exams [5–9]. These question banks typically offer the opportunity to review questions in “tutor mode,” with direct feedback following each question. Some have proposed the value of this format to augment learning points from the question explanation through closely aligning them to the question stem [2]. Both formats encourage content retrieval which has been shown to enhance learning by forming memories that are more durable and flexible in future application. Additionally, the direct feedback helps the learner establish what they know (answer correctly) and need to learn (answer incorrectly), thus allowing them to prioritize their study time. This can be particularly helpful during compressed study times frequent in medical education [10].

We developed a microbiology question bank that delivers content in format similar to “tutor mode” and is enhanced with gamification and clinical pearls to supplement our microbiology course curriculum. Gamification in its simplest terms is applying game elements to nongame context such as an education activity. This tool when used purposely can engage and motivate learners through extrinsic motivators (such as prizes and leaderboards) as well as intrinsic motivators (such as teamwork and challenges) [5, 11–13]. Our question bank operates through a locally developed education smart phone software application for ease of use (Kaizen Education) [12, 14]. We hypothesized the game would engage learners and increase their knowledge base as demonstrated through improved microbiology examination scores.

## Methods

### Setting

Our curriculum was implemented within the Microbiology module during the first semester of medical school at the University of Alabama School of Medicine for the matriculating class of 2019. Our average class size is 186 students. The first semester lasts 4 months and includes multiple modules representing fundamental areas (physiology, biochemistry, histology, pharmacology, pathology, genetics, immunology, and microbiology) which provide the foundation for an organ module–based curriculum that continues through the remaining pre-clerkship years. The Microbiology module lasts for 3 weeks at the end of the first semester of the first year of medical school. Subsequently, microbiology content is integrated into each successive organ module via various didactics for the remainder of the pre-clerkship curriculum. This study was determined exempt by the Institutional Review Board (IRB) at the University of Alabama at Birmingham.

### Gamification, Software, and Educational Competition Design

The Kaizen Education software was developed at our institution in partnership with our National Institutes of Health (NIH)–Funded Center for Clinical and Translational Science Award (CTSA). It has been used across many disciplines including graduate medical education, dentistry, undergraduate medical education, and to train in research methodology [12, 15–17]. The platform consists of two parts: a free, mobile application that users can download onto their personal device (iOS, Android, or web access available) and an online Game Manager Portal that guides educators through the creation of games.

A teaching fellow (J.W.) created 56 multiple choice questions based off course content, 57% were written in a clinical vignette style, and the remaining were recall questions aimed at recognizing core concepts. The course directors who constructed the exam (M.P. and R.L.) approved of the content areas and a sample of the questions but limited their participation in the question creation to preserve integrity of the exam. The questions were released at 00:15 on weekdays and addressed topics covered in didactics scheduled for that calendar day. Upon answering a question within the app, students receive immediate feedback that includes an explanation of the core concept, a clinically focused fact related to the concept (a “clinical pearl”), and an explanation highlighting why the remaining answer options were incorrect. Each question and explanation could include text, video, images, or audio to underscore teaching points. Review questions were released on weekend days and the last 2 days of the module featured large blocks of review questions to prepare for the final exam. Three days were selected throughout the competition as “timed” days. There were 16 questions (28%) released on these days and each had a timer of 120 s that began when the question was opened. Answering correctly within the time limit earned double points, while answering correctly outside the time limit would still earn the typical point value. The competition portion ended 48 h before the final exam, but all questions were accessible at any time from release through the end of the course.

Students played as individuals and also as part of a team coinciding with their learning communities, a longitudinal program for our students where they compete to achieve the highest total of “cup” points each year. The winning team received 25 points to count toward that year’s cup competition. Individually, students received points for correct answers and scores were viewable on a leaderboard within the application. The students had the opportunity to change their username to an alias for anonymity within visible display of the application. In addition, in-game badges were awarded for achieving pre-determined point totals (Level badges), getting several answers correct in a row (Hot-streak badges), and completing questions on the day of release on consecutive days (Marathon badges). The application allows for students to communicate within their team or with the game creator to challenge questions or ask for clarification. We initiated communication with the participating students through the application twice per week to offer game updates and highlight those in the lead. Finally, participation in any portion of the game was voluntary.

### Data and Statistical Analysis

The application collects data on all user interactions with the software including: questions completed, days logged into application, correct answers, time to answer, and badges earned. This data was not available until several weeks after the course was completed and course grades finalized. At this time, the individual user data was connected to course grades and de-identified by a statistician (J.C.) who had no direct involvement with the students. We completed descriptive analyses of learner utilization of the Kaizen Education software with these data. We measured “player efficiency rating” (PER), a composite measure of player accuracy, questions answered, and time to response [14, 17]. Answering questions correctly, answering more questions, and answering questions in a timely manner lead to a higher PER. Specifically PER was calculated by awarding 1 point for each correct answer that occurred before the exam date corresponding to the question release; no points were awarded for incorrect answers or those answered later in the course. Additional points were awarded for each question answered longitudinally (0.25 points for questions 10–20, 0.5 points for questions 21–40, and 0.75 points for questions 41–56). Finally, players received an additional 0.5 points for answering questions on the day of release and 0.25 within 1 week of release. The 8 students who did not participate in Kaizen were given a PER score of 0 and included in subsequent analysis. Individual student’s mean exam grades had a positive correlation with continuous PER by Spearman’s rank correlation. We then explored PER tertiles compared using median exam average by the Kruskal–Wallis test to identify the group that may have benefited most. We also provided an optional and anonymous survey through Google Forms at the end of the module focused on their experience with the game. The survey consisted of a validated 10-item System Usability Scale (SUS) [18], and a locally developed 7-item survey (5 point Likert scale of strongly disagree to strongly agree) created to elicit perceptions of Kaizen’s effect on learning core microbiology. “Disagree and strongly disagree” were combined for ease in reporting.

## Results

**Fig. 1:**
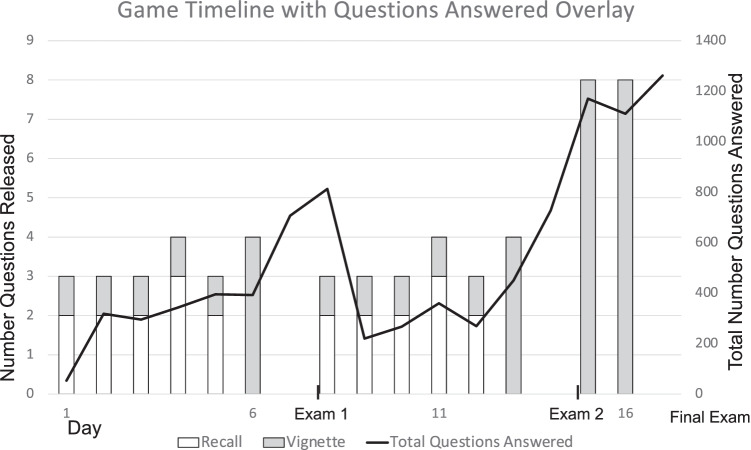
Game timeline with questions answered overlay

One hundred eighty-one of 189 total medical students enrolled in the course participated in Kaizen and 96% of total questions released were answered (Table 1). The average number of users accessing the app on any given day throughout all exam periods ranged from 60 to 65. However, we observed an upsurge in questions answered in the 24 h before each exam and during the 48 h before the competition ended (Fig. 1). Overall question accuracy was 82.6%. A higher accuracy was seen for vignette (83.8%) compared to recall (81.0%) *p* = 0.0006 and for untimed (84.7%) compared to timed (77.2%) *p* = < 0.0001. The mean PER was 84.75 and reflects the high engagement and accuracy observed.

**Table 1:** Game demographics

Characteristic	Result
Students who logged into game (% total class)	181/189 (95.8%)
Days played (median (Q1, Q3))	6 (4, 9)
Total questions answered	96.4%
Available questions answered before exam 1	83.5%
Available questions answered before exam 2	79.6%
Badges earned (median (Q1, Q3))	9 (8, 10)
Number of Daily Average User before exam 1	65.00
Number of Daily Average User before exam 2	60.29
Number of Daily Average User overall	61.74
Overall question accuracy	82.59%
Vignette questions accuracy	83.66%*
Recall questions accuracy	80.98%*
Timed questions accuracy^**^	77.20%*
Non-timed questions accuracy	84.66%*
Player efficiency rating (median, (Q1, Q3) interquartile range)	84.75 (67.75, 90.00)

^*^A significant difference was found between vignette and recall question accuracy (*p* = 0.0006) and timed and untimed question accuracy (*p* ≤ 0.0001); ^**^Timed questions were awarded double points if answered within 120 s

A total of 61 (34%) participants responded to the end of the course survey (Table 2). The app scored highly on the SUS (System Usability Scale) at 87%, which is within the highest range for each component of the score. Over 80% of survey respondents indicated that participation improved their performance in the course, helped them prioritize concepts for review, that it helped them prepare for quizzes, identify gaps in their knowledge, and enhanced their application of basic science knowledge and concepts to clinical scenarios (Table 2). The major themes from the student feedback included appreciation of the game content, practice questions, and explanations. Six students commented about the importance of connecting basic science to clinical medicine. “The clinical scenarios testing basic science knowledge tied everything together” and “(Kaizen) forced me to make clinical correlations with the material presented in lecture.” We surveyed aspects of gamification which the majority found competition beneficial to learning (61%) and teamwork increased engagement (58%) (Table 2). A handful of comments addressed this as well and included “Kaizen was fun, bite size learning” and “Even though the competition did not enhance my learning, I still loved it! It was fun and made learning feel light-hearted.”

**Table 2:** Survey responses

Survey question (*n* = 61)	SA Strongly Agree	A Agree	N Neutral	D Disagree
My performance in the class was improved by Kaizen *n* (%)	25 (41%)	25 (41%)	9 (15%)	2 (3%)
Kaizen helped me prioritize concepts for review *n* (%)	28 (46%)	22 (36%)	9 (15%)	2 (3%)
Kaizen helped me prepare for quizzes *n* (%)	29 (48%)	21 (34%)	9 (15%)	3 (5%)
Kaizen helped me identify gaps in my knowledge *n* (%)	32 (51%)	25 (41%)	0 (0%)	4 (6%)
Kaizen forced me to apply theoretical knowledge to clinical scenarios *n* (%)	33 (54%)	24 (39%)	0 (0%)	4 (6%)
The competitive aspects of Kaizen were beneficial to my learning *n* (%)	19 (31%)	18 (30%)	16 (26%)	8 (13%)
The team engagement increased my participation in Kaizen *n* (%)	20 (33%)	15 (25%)	16 (26%)	10 (16%)

**Fig. 2:**
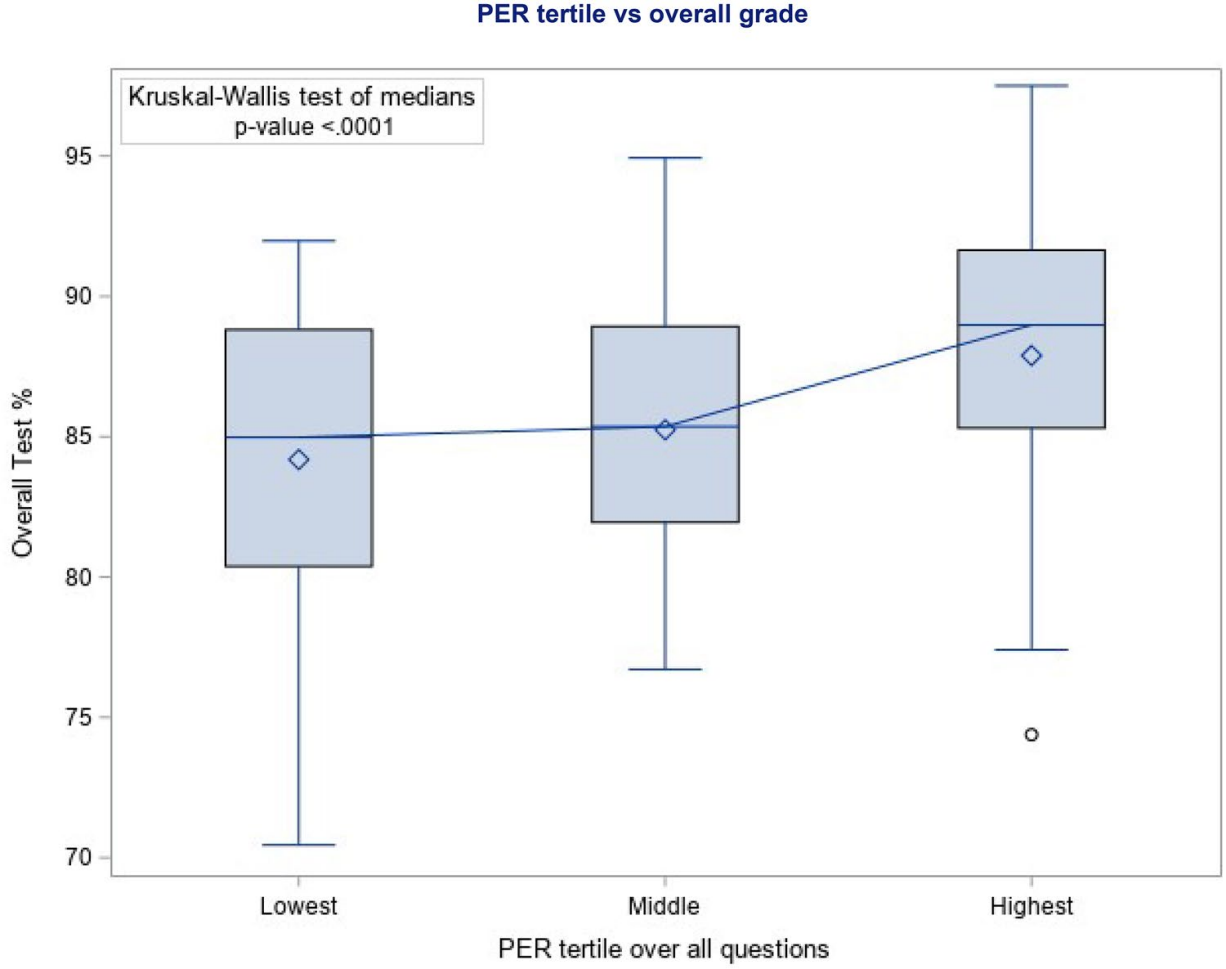
Overall exam average by PER tertile

Individual student’s mean exam grades demonstrated a modest positive correlation with continuous PER by Spearman’s rank correlation, 0.338 (*p*-value < 0.001). The KW test found a statistically significant difference between the average exam scores of the player groups when divided into tertiles based on PER (*p* = 0.0002) (Fig. 2). When comparing pair-wise, there was a statistically significant difference between the highest and lowest (0.0004) tertiles, as well as the highest and middle (0.0045) tertiles. The lower two tertiles were not significantly different (0.73). The students who did not participate were evenly split by mean exam score.

## Discussion

Several studies have investigated addition of gamification into UME with mixed results [5, 6, 11–13]. Our study is the first to our knowledge to investigate its application within a UME microbiology curriculum. Our strengths include the high engagement by a large number of students and utilization of a teaching fellow to provide clinical pearls. A critique of prior studies has been the limitation in comparative groups, driven in part by the need for an equal experience for students within their core curriculum [6, 13]. Completion of Kaizen microbiology questions was introduced as an optional supplement to our curriculum that achieved excellent participation overall, with 87% of students completing all questions. This high participation limits our control group, and thus we incorporated PER to differentiate engagement levels. We found that those in the highest PER tertile group also had higher average exam scores, demonstrating an association between engagement in a gamified microbiology education question bank and higher course performance.

Our survey indicates a positive user experience with the Kaizen software with a system usability score (SUS) well above the industry accepted average [18]. Overall, our game was rated favorably with the highest positive ratings to statements that Kaizen microbiology “helped identify gaps in knowledge,” “forced me to apply theoretical knowledge to clinical scenarios,” and “made it easy to learn and retain microbiology knowledge.” Despite the short time available to our course within the first-year curriculum, we found students engaged with this optional supplementary activity delivered outside of the typical teaching space. The high participation and survey response suggest the delivery format was enjoyed and enhanced their perceived microbiology learning experience.

Our questions emphasized foundational basic science concepts and embedded these concepts in clinical context. The utility of supplementary question banks within medical education has been outlined previously [5, 9, 19, 20]. We provided detailed answer explanations with “clinical pearls” to connect core knowledge to clinical infectious diseases. Involving an Infectious Diseases fellow to develop the question content and serve as game manager allowed for their development as an educator while reinforcing the clinical applicability of the content, although all questions were delivered with immediate feedback following there was variation in type (recall and vignette) and time restriction (timed vs untimed). We did find a significant difference between these question styles with vignette and untimed questions having a slight (2.8–7.5%) yet statistical difference in accuracy. It is not surprising that restricting the time limit led to decreased accuracy, restricting time to access additional resources and simulating a testing environment. As the last fundamentals course before organ modules, students are still becoming accustomed to clinical vignette style questions. The difference in accuracy may simply reflect that those types of questions were written with a lower difficulty level than a recall question. The benefits of various styles of questions and delivery to learning is an area worthy of further research.

The ongoing COVID-19 pandemic has highlighted the need for innovative methods to enhance distance learning activities and technologies that foster student engagement. As gamification use expands within medical education, there is a need to understand which of its various components are most successful. Concerns have been raised that when not balanced, a focus on extrinsic motivation may negatively impact long-term learning [6, 11]. While our study design was not able to investigate the impact of each individual intrinsic and extrinsic motivator applied to our educational game directly, a total of 60% and 56% agreed or strongly agreed, respectively, that competition and team assignment components of the game were beneficial. These survey components of gamification were not as uniformly positive as perceived benefit of learning content; however, it is expected that some would be more motivated by the extrinsic rewards such as the competition with leaderboard and points, while others are motivated by the intrinsic rewards of competing as a team and marathon badges. These game elements serve to augment the return to the question bank content which is itself rich in intrinsic rewards of problem solving, immediate feedback, as well as learning mechanics of retrieval and pre-testing [21].

Our study has several limitations. Most notably, it was conducted in a single year within a single institution and curriculum, with instructors and game developers working collaboratively. Although the core microbiology material is similar across medical schools within the USA, curriculums vary and course examinations may emphasize various combinations of select material [22]. The ability to introduce additional parallel educational content augmented with gamification should be possible across any curricular design, but games with a longer timeline may have challenges in longitudinal student retention. Our survey response rate was low, which we attributed to survey fatigue, a common issue among our medical students, and timing immediately before winter break. As an observational study, we only report association between higher PER values and higher exam scores. Study habits, prior microbiology experience, use of other supplemental material, and comfort with multiple choice question formats may confound engagement and accuracy in this supplemental curriculum. However, the Kaizen-Micro game leverages external motivators, delivers questions at pace dictated by the student user, and allows use of outside resources which may create a more student-driven pace than the typical course-work and testing timeline. Additionally, medical students as a whole are a select group of highly motivated and efficient learners, whose use of study resources, including question banks, has been documented extensively (4, 7, 8). Finally, this educational tool was resource intensive in both creating the accompanying questions, explanations, and game design. Collectively, we estimate educator preparation time for the single game described in this study at approximately 30–40 h. Fortunately, questions can be easily updated and implemented in subsequent years, while the user interface for the game manager tools is continually refined by the development team in response to user feedback. This game has been delivered in subsequent years with limited modifications required. We would like to note that while we chose to use a locally produced electronic platform (Kaizen Education), there are several additional free resources that use elements of gamification and could replicate a similar format [5, 20, 23, 24].

We found that implementation of a software-based multiple choice question–based knowledge competition, augmented with gamification, led to high student engagement and was associated with higher average exam scores among the most highly engaged users. The students perceived the activity as being beneficial to learning core microbiology as well as connecting the concepts to the clinical realm. Our study employed gamification and technology to enhance curriculum, and we encourage educators to utilize innovative instructional tools to maintain learner engagement during core pre-clerkship content areas such as microbiology education.
